# Disease-modifying effects of metabolic perturbations in ALS/FTLD

**DOI:** 10.1186/s13024-018-0294-0

**Published:** 2018-12-04

**Authors:** Ali Jawaid, Romesa Khan, Magdalini Polymenidou, Paul E. Schulz

**Affiliations:** 1 Laboratory of Neuroepigenetics, Brain Research Institute, University of Zurich (UZH)/ Swiss Federal Institute of Technology (ETH), Winterthurerstr. 190, 8057 Zurich, Switzerland; 2 Syed Babar Ali School of Science and Engineering (SBASSE), Lahore University of Management Sciences (LUMS), Lahore, Pakistan; 3 Institute of Molecular Life Sciences, University of Zurich, Zurich, Switzerland; 4 Department of Neurology, The McGovern Medical School of UT Health, Houston, TX USA

## Abstract

Amyotrophic lateral sclerosis (ALS) and frontotemporal lobar degeneration (FTLD) are two fatal neurodegenerative disorders with considerable clinical, pathological and genetic overlap. Both disorders are characterized by the accumulation of pathological protein aggregates that contain a number of proteins, most notably TAR DNA binding protein 43 kDa (TDP-43). Surprisingly, recent clinical studies suggest that dyslipidemia, high body mass index, and type 2 diabetes mellitus are associated with better clinical outcomes in ALS. Moreover, ALS and FTLD patients have a significantly lower incidence of cardiovascular disease, supporting the idea that an unfavorable metabolic profile may be beneficial in ALS and FTLD. The two most widely studied ALS/FTLD models, super-oxide dismutase 1 (SOD1) and TAR DNA binding protein of 43 kDA (TDP-43), reveal metabolic dysfunction and a positive effect of metabolic strategies on disease onset and/or progression. In addition, molecular studies reveal a role for ALS/FTLD-associated proteins in the regulation of cellular and whole-body metabolism. Here, we systematically evaluate these observations and discuss how changes in cellular glucose/lipid metabolism may result in abnormal protein aggregations in ALS and FTLD, which may have important implications for new treatment strategies for ALS/FTLD and possibly other neurodegenerative conditions.

## Background

Amyotrophic lateral sclerosis (ALS) is a fatal neurodegenerative disorder that is characterized by the progressive degeneration of both upper and lower motor neurons, which results in a multitude of motor symptoms, including muscle weakness, fasciculations, spasticity, dysphagia, and eventually respiratory dysfunction [167]. There is also an established clinical overlap between ALS and frontotemporal lobar degeneration (FTLD). FTLD is a pathological diagnosis that manifests clinically in the form of frontotemporal dementia (FTD), which is characterized by cognitive, behavioral, and linguistic dysfunction. Almost 50% of ALS patients show cognitive impairment of the type observed in FTD, with 15% of ALS cases meeting diagnostic criteria for FTD at the time ALS is diagnosed [144]. In addition, 15% of FTLD cases have clinically detectable motor symptoms [92]. Ten percent of all ALS patients, and one third of all FTLD patients, have a positive family history with at least one immediate family member having the disease [92, 119]. The prevalence (62%) and pattern (related to FTD) of cognitive impairment in familial ALS are similar to that of sporadic ALS [183].

Like many neurodegenerative diseases, ALS and FTLD are associated with the abnormal aggregation of specific proteins in neurons and glia. Superoxide dismutase 1 (SOD1) was the first aggregated protein to be associated with ALS, just over two decades ago [147]. Since then, several other proteins have been shown to form abnormal neuronal aggregates in ALS and FTLD, including TAR DNA binding protein 43 kDa (TDP-43) and fused in sarcoma (FUS). According to the predominant type of aggregated protein found, ALS is classified as ALS-TDP, ALS-FUS, or ALS-SOD; and FTLD as FTLD-Tau, FTLD-TDP, FTLD-FUS, or FTLD-UPS (for Ubiquitin-Proteasome system) [102]. Among these subtypes, ALS-TDP and FTLD-TDP are the most common, representing 98% of ALS and 45% of FTLD cases, and are the most important indicator that there is a pathophysiological continuum between the two disorders [92].

The molecular mechanisms that underlie abnormal neuronal protein aggregation in ALS/FTLD remain unknown, but mutations in several genes can trigger the formation of such aggregates. TDP-43 aggregates result from mutations in the *TARDBP* gene, as well as from mutations in progranulin (*PGRN*), chromosome 9 open reading frame 72 (*C9ORF72*), valosin-containing protein (*VCP*), p62/sequestosome (*SQSTM1*), optineurin (*OPTN*), chromatin modifying protein 2B (*CHMP2B*), and Ubiquilin 2 (*UBQLN2*). Similarly, FUS protein aggregates result from mutations in the *FUS* gene, as well as in TATA-binding protein-associated factor 15 (*TAF15*), and Ewing’s sarcoma breakpoint region 1 (*EWSR1*) [101]. Notably, abnormal aggregates are present in all ALS/FTLD cases even when no genetic mutations are present. As discussed later, the causes of neuronal death in ALS/FTLD are also unknown and are being investigated in animal models, with studies supporting a role for oxidative damage, excitotoxicity, and apoptosis [46, 110, 132, 170].

Clinical and epidemiological studies have identified a number of risk factors and disease-modifiers that can impact the clinical course in ALS and FTLD, including metabolic parameters. Notably, a conventionally ‘risky’ cardiovascular profile, such as a high body mass index (BMI), or diabetes mellitus type 2, might protect individuals from ALS by delaying the onset of symptoms and/ or slowing clinical progression, whereas, a ‘beneficial’ cardiovascular profile, with a low body mass index, an athletic lifestyle, and low blood cholesterol levels, may increase the risk or worsen the prognosis [77]. This intriguing relationship between metabolism, ALS and FTLD warrants a thorough evaluation of the rapidly accumulating clinical evidence on the topic, as well as, exploration of molecular data that could reveal their biological underpinnings and suggest new treatment strategies.

In this review, we discuss the disease-modifying effects of metabolic disorders in ALS/FTLD and the preclinical studies that demonstrate the involvement of ALS- and FTLD-associated genes/proteins in metabolic pathways. Moreover, we present several hypotheses as to how metabolic perturbations may modulate pathological protein aggregation or its consequences in ALS and FTLD. We conclude by discussing a potential disease-modifying effect of metabolic disorders in other neurodegenerative disorders.

## The disease-modifying effects of metabolic disorders in ALS

Table 1 summarizes studies suggesting a protective role for traditionally “risky” cardiovascular profiles in ALS and FTLD.

**Table 1:** Clinical studies finding associations between metabolic diseases or risk factors and ALS/ FTLD risk or prognosis

Metabolic condition	Evidence supporting a beneficial effect on risk/prognosis of ALS	Evidence not supporting a beneficial effect on risk/ prognosis of ALS	Citation, sample size (n)
Dyslipidemia	Increased survival in ALS by 12.5 months	N/A	Dupuis et al. [47] (*n* = 369 ALS, *n* = 286 controls)
	Increased survival in ALS by 14 months		Dorst et al. [44] (*n* = 488 ALS)
	Increased survival in ALS by 5.8 months		Huang et al. [70] (*n* = 413 ALS)
	Delay in ALS onset by almost 6 years		Hollinger et al. [67] (*n* = 1439 ALS)
	N/A	Increased survival in ALS confounded by BMI	Paganoni et al. [124] (*n* = 427 ALS)
		Increased survival in ALS confounded by age and BMI	Rafiq et al. [140] (*n* = 512 ALS)
		Increased survival in ALS not significant	Dedic et al. [35] (*n* = 82 ALS)
T2DM	Delay in ALS onset by 4 years	N/A	Jawaid et al. [75, 76] (*n* = 2371 ALS)
	Decreased risk of ALS in non-insulin dependent T2DM		Mariosa et al. [105] (*n* = 5108 ALS, *n* = 25,540 controls)
	Decreased prevalence of T2DM in ALS		Paganoni et al. [125]
	Decreased prevalence of T2DM in ALS		Mitchell et al. [111] (*n* = 1288 ALS, *n* = 7561 controls)
	Decreased risk of ALS in people with DM		D’Ovidio et al. [32] (*n* = 727,977)
	N/A	No association between T2DM and ALS risk	Sun et al. [165] (*n* = 615,492 diabetics, *n* = 614,835 controls)
		Increased mortality in ALS patients with high baseline HbA1c	Wei et al. [182] (*n* = 450 ALS)
High BMI	Increase in BMI slowed functional decline in ALS	N/A	Jawaid et al. [75, 76] (*n* = 285 ALS)
	High BMI slowed progression and decreased mortality in ALS		Gallo et al. [56] (*n* = 518,108 over-all) Reich-Slotky et al. [141] (*n* = 150 ALS)
Cardiovascular diseases	Decreased risk of FTLD	N/A	Kalkonde et al. [80] (*n* = 63 FTD, *n* = 491 non-FTD dementias)

*Abbreviations*: *BMI* body mass index, *T2DM* type 2 diabetes mellitus, *N/A* not applicable

### Dyslipidemia

Dyslipidemia appears to be protective in ALS, both in terms of improved prognosis and reduced risk of the disease. An abnormally high low-density lipoprotein (LDL)/high-density lipoprotein (HDL) ratio is associated with an increase in survival by 12 months in ALS [*n* = 369, [47]]. Similarly, triglycerides above 1.47 mmol/L prolong survival in ALS patients by 14 months [*n* = 488, [44]]. Furthermore, antecedent hyperlipidemia delays ALS onset [*n* = 1439, [67]]. This association is further supported by studies demonstrating that a conventionally ‘favorable’ vascular profile, including hypolipidemia, increase the risk of ALS. Sometimes, this in a gender-dependent fashion with only male ALS patients having significantly lower total cholesterol and triglycerides than male controls [166, 191].

The effect of dyslipidemia on ALS survival could be confounded by other factors, such as body mass index [BMI, [124]]. Rafiq et al. showed a beneficial effect of dyslipidemia on survival in a cohort of ALS patients (*n* = 512) enrolled in the Olesoxime (a neuroprotective compound) phase II-III clinical trial for ALS treatment; however, the statistical significance was lost when adjusted for age of onset, or BMI [140]. Similarly, Dedic et al. observed an approximately 8 month longer survival in ALS patients (*n* = 82) who have dyslipidemia compared to those without dyslipidemia, but it was not statistically significant [35]. Both studies, however, included a remarkably higher proportion of ALS patient with dyslipidemia (64% in Rafiq et al. and 52% in Dedic et al.) than has been reported for other ALS cohorts (around 40% in Hollinger et al.), raising a question about the generalizability of these results.

### Diabetes mellitus

Some earlier reports suggested that ALS patients might have certain features of diabetes mellitus (DM), such as glucose intolerance [143]. More recent studies have shown a considerate influence of diabetes mellitus on ALS risk and prognosis. Insulin-dependent Type 1 DM (T1DM) appears to increase the risk of ALS 5.83 times (OR: 1.87–15.51, [105]). In stark contrast, however, Type 2 DM (T2DM), a form of insulin-independent DM, appears to have a beneficial effect on the course of ALS in terms of a reduced risk and better prognosis [74, 77]. A likely explanation for contrasting effects of T1DM and T2DM on ALS could be the different etiopathogenesis of the diseases. In contrast to TD2M, T1DM is an autoimmune disorder, and they are known to increase the risk of ALS [171].

A Swedish ALS cohort (*n* = 5108) showed an inverse association (OR: 0.66, 95% CI 0.53–0.81) between non-insulin dependent DM (likely T2DM) and the risk of ALS [105]. Two different American cohorts have also reported a reduced prevalence of T2DM in ALS patients, suggesting that T2DM might reduce the risk of ALS [111, 125]. In our large study of 2371 patients, pre-morbid T2DM, i.e., occurring before onset of ALS symptoms, was associated with a four-year delay in ALS onset and a prolonged survival of about six months [75]. Another study showed a trend (*p* < 0.1) towards slower disease progression in ALS patients with pre-morbid T2DM [74, 125]. Finally, a recent study investigated for ALS occurrence in all residents of Turin (*n* = 727,977) who were 14 years of age at 1996 during an assessment period from 1998 to 2014. During this period, 397 patients developed ALS and diabetes was found to decrease the risk (hazard ratio: 0.30, 95% CI 0.19–0.45) of ALS occurrence [32]. A Taiwanese study, however, did not show a link between T2DM and ALS [165]. Similarly, an assessment of the association with baseline HbA1c and survival in Chinese ALS patients (*n* = 450) showed that increased baseline HbA1c was associated with increased risk of mortality [182]. These discrepant results in Asian ALS patients raise the possibility that the protective effect of T2DM on ALS could depend on ethnicity [97].

### Body weight

The association between body weight and ALS is very interesting. Several studies have shown that a low (under-weight) pre-morbid body mass index (BMI) is a risk factor for ALS ([72, 104, 116, 122]). At the other end of the spectrum, several studies have shown that a high (over-weight or obese) BMI at diagnosis is associated with slower disease progression and decreased mortality in ALS [56, 141]. In another ALS cohort (*n* = 285), the rate of symptom progression was inversely related to changes in BMI during the first year after ALS diagnosis [76]. Other studies have also demonstrated that a decrease in BMI after onset of motor symptoms dramatically reduces survival in ALS patients [125, 157]. Collectively, these studies strongly suggest an association between lower body weight and ALS risk, and a worse prognosis in patients with faster weight loss after ALS diagnosis.

### Physical activity

Physical activity is an important determinant of metabolic homeostasis and hence may be very relevant to ALS risk. Several studies, in fact, report that high levels of physical activity and/or an athletic lifestyle significantly increase the risk and/or worsen the prognosis of ALS [11, 25, 72, 98, 123, 169]. However, not all studies have come to this conclusion [173, 177], and one study suggests that exercise and high levels of physical activity may even be beneficial, with overall physical activity associated with a reduced odds (OR = 0.56, 95% CI: 0.36–0.87) of having ALS [138].

It is possible that this wide variability in outcomes is related to the fact that almost all of these studies have been retrospective. A recent Cochrane systematic review on the role of exercise on ALS risk and progression identified a lack of comparable outcome parameters in retrospective studies, as well as a lack of prospective analyses as likely reasons for these conflicting results [33]. The first prospective study (*n* = 472,100) reported a borderline significantly (*p* = 0.042) decreased risk of dying (HR = 0.67, 95% CI: 0.42–1.06) from ALS in patients with high levels of physical exercise at the time of enrollment [55]. However, a robust case-control study of ALS patients (*n* = 1557) vs. matched controls (*n* = 2992) recruited from five population-based registries in The Netherlands, Ireland, and Italy revealed a modest linear association between physical activity and ALS risk ((OR: 1.06, *p* < 0.001), [179]). More prospective analyses in future will likely clarify the association between exercise and ALS.

### Metabolic drugs

Clinical studies suggest that there is an intriguing relationship between the use of drugs that treat common metabolic conditions and the risk and rate of progression of ALS.

Despite rapidly emerging evidence that T2DM is protective in ALS, certain anti-diabetic drugs have been used to treat ALS because of their anti-inflammatory properties. Pioglitazone, which is used to manage T2DM, enhanced the survival of a mouse SOD1 model of ALS; however, a phase II clinical trial suggested that this drug increased the risk of death in ALS patients, although this increase lacked statistical significance [48, 77]). Collectively, the results of these studies suggest caution in use of drugs that are used to treat T2DM and dyslipidemia in ALS patients.

Preliminary reports have suggested an increased occurrence of ALS among users of statins, which are used to treat dyslipidemia [50]. However, more rigorous analyses have subsequently found a null association between statins and ALS risk (OR: 0.96, 95% CI: 0.73–1.28, [158]), as well as, prognosis [45]. Similarly, other reports suggest that the association between statin use and risk of ALS could be gender-dependent, with only female ALS patients on statins showing a significantly faster decline (a decline of 3 points more over 12 months) in (ALS-functional rating scale) ALS-FRS compared to those not on statins [118].

### Diet-based trials for ALS treatment

The evidence that changes in BMI and metabolic perturbations can alter the course of ALS has led to several trials of diet-based treatment for this disease. A notable example is a prospective interventional study (*n* = 26) that compared a high-fat and high-carbohydrate diet in ALS, which showed that both diets could stabilize or increase the BMI of ALS patients [43]. However, this study did not explore the effects of a stabilized BMI on disease progression or survival. In another placebo-controlled, phase II trial (*n* = 26), mortality and disease progression in ALS patients on a high calorie/high carbohydrate (HC/HC) diet or on a high-calorie/high-fat (HC/HF) diet was compared to mortality and disease progression in ALS patients on a normal control diet. This trial found significantly (*p* < 0.03) reduced mortality (*p* < 0.03) and a trend towards slower ALS progression (*p* = 0.07), in the HC/HC group versus controls [185].

Other studies also suggest beneficial effects (slower progression and/ or longer survival) of nutritional counseling [6], and of diets that contain omega-3 fatty acids [53], anti-oxidants and carotenes [78, 121], acetyl carnitine [12], and Vitamin E [54] in ALS. The role of Vitamin D, however, remains unclear, with both its deficiency [20, 82] and increased levels (-[15]) having been associated with worse prognosis in ALS.

In conclusion, current evidence strongly supports a role for dyslipidemia, T2DM, and obesity in reducing the risk for acquiring ALS and improving its prognosis. A recent study further showed an inverse association between serum retinol binding protein-4 and ALS risk and prognosis. Serum retinol binding protein 4 indicates the overlapping molecular manisfestaion of insulin resistance in dyslipidemia/T2DM/obesity, and thus provides further evidence for a protective effect of metabolic disorders in ALS [148]. The evidence supporting a disease-modifying effect of exercise and dietary regimens on ALS is, however, less conclusive and warrants further investigations. There is also evidence for a disease-modifying role of metabolic disorders on the risk and prognosis of the closely related disorder, FTLD.

## The disease-modifying effect of metabolic disorders in FTLD

The disease-modifying effects of metabolic disorders in FTLD have not been well studied. However, in a large cohort of military veterans with dementia (*n* = 554), the risk of developing FTD was reduced (OR = 0.4, CI = 0.3–0.96) in patients with cardiovascular disease [80]. A study of 31 behavioral variant FTD patients and 19 controls found that behavioral FTD patients have considerably higher (1.9 mmol/L) serum triglycerides than controls (0.85 mmol/L), which is a known cardiovascular risk factor [3].

More studies are thus needed to ascertain whether metabolic disorders have a protective effect on the progression of FTLD, similar to their effect on ALS. It is also plausible that metabolic perturbations have contrasting effects on the motor and cognitive functioning of the brain. We found, for example, that T2DM has contrasting effects on the motor and cognitive functions of patients with ALS. Pre-morbid T2DM delayed the onset of motor symptoms in ALS patients, but impaired frontal and temporal cognitive functioning [75]. Animal models and neuropathological studies will certainly help delineate these effects.

### Evidence for metabolic dysfunction in ALS/ FTLD through omics and biochemical assays

Mass-spectrometry based omics approaches present a great opportunity for a wide-scale and robust investigation of a pathophysiological link between neurodegenerative and metabolic processes in ALS/ FTLD. An initial metabolomics-based comparison between ALS patients (*n* = 19) and controls (*n* = 33) found an increase in 6 metabolites in the plasma of ALS patients, however the study did not indicate their exact characteristics [149]. Another study showed an increase in 23 metabolites, notably related to hypermetabolism, oxidative damage, and mitochondrial dysfunction in the plasma of ALS patients (*n* = 62 + 99 for two different assessments) compared to controls (*n* = 69 + 48 for two different assessments) [86]. Importantly, a number of these metabolites, such as Carnitine and Paraxanthine are also altered in T2DM and dyslipidemia, in the opposite direction [13, 172]. A third study demonstrated changes in plasma tryptophan, arginine, and proline metabolism pathways in the plasma of ALS patients (*n* = 24) relative to age- and gender-matched controls (*n* = 24) [134]. Intriguingly, some of these metabolites, such as Valine and Serine, again show an opposite expression in dyslipidemia and T2DM [22, 113, 189]. More recent lipidomics analyses further reveal hypoalphalipoproteinemia and increased CSF phosphatidylcholine as metabolic signatures of bvFTD [84] and ALS [16] respectively.

In addition to metabolomics, other biochemical analyses have also revealed peripheral metabolic signatures unique to ALS. A study on peripheral lipoproteins revealed a characteristic low IDL-B and high LDL-1 in the serum of ALS patients [37]. Another study, which quantified 40 different sterols, showed that (25R) 26-hydroxycholesterol, the immediate precursor of 3β-hydroxycholest-5-en-26-oic acid, was reduced in ALS patients compared with controls [2]. These analyses are very important additions to the aforementioned clinical observations showing the effect of metabolic disorders on ALS/ FTLD.

## Metabolic dysfunction in animal models of ALS/FTLD

Another important approach to understanding and utilizing metabolic changes in ASL/FTD are animal models where one can control for many variables that are challenging in humans.

SOD1 and TDP-43 are the most widely studied animal models of ALS/FTLD, and there are several important recent studies manipulating SOD1 and TDP-43 genes (knock-out, conditional knock-out, knock-in, over-expression of the endogenous or human protein, mutations) to decipher metabolic changes in these animals and utilizing these findings for pre-clinical trials of metabolism-based therapeutic strategies.

### Metabolic dysfunction in the SOD1 model of ALS

Missense mutations in the Cu/Zn-binding superoxide dismutase (*SOD1*) gene, which encodes a ubiquitously expressed antioxidant enzyme, are reported in 10–20% cases of familial ALS [38, 147]. *Sod1* mutant mice develop many pathological features of human ALS, including loss of upper and lower motor neurons, progressive paralysis, and the accumulation of ubiquitinated inclusions in the neurons that contain misfolded Sod1 [59, 145].

SOD1 mice also exhibit signs of metabolic dysfunction, including increased energy expenditures, skeletal muscle hypermetabolism, and reduced adipose tissue, even at the asymptomatic stage [49, 127]. This is followed by rapid weight loss after symptom onset [62]. Furthermore, *Sod1* mutant mice also exhibit wide-ranging deregulation of their lipid metabolism, including peripheral hypolipidemia [41, 52, 83] and upregulated levels of ceramides, glucosylceramides and glycosphingolipids [42, 64, 155].

The metabolic perturbations observed in *Sod1* mutant mice have prompted scientists to explore their therapeutic potential. The administration of a high-fat, energy-rich diet protected *Sod1* mouse mutants against motor disease progression [49, 52]. Similarly mitigating effects have been reported following the treatment of *Sod1* mouse mutants with the triglyceride triheptanoin [168] or with a ganglioside, GM3 [42]. Switching mutant *Sod1* mice from a normal to a ketogenic diet, or giving them caprylic triglyceride, which is metabolized to ketone bodies, restored their mitochondrial ATP production and was accompanied by reduced neuronal loss, delayed onset of motor dysfunction, and enhanced survival [193, 194]. Arginine-alpha- ketoglutarate supplementation also improved the motor functions and lifespan of *Sod1* mutant mice [7]. In contrast, caloric restriction in rodent models expressing mutant Sod1, have shown detrimental effects, i.e. earlier symptom onset and shortened lifespan [60, 61, 133].

### Metabolic dysfunction in TDP-43 models

The presence of ubiquitinated and phosphorylated cytoplasmic inclusions of TDP-43 in the CNS of patients with ALS and FTLD [119], and the subsequent identification of *TARDBP* mutations in the sporadic and familial forms of these diseases [57, 79, 160, 175, 192], have led to the rapid development of TDP-43 transgenic models. Mutant *Tardbp* mice manifest some key features of human ALS and FTLD, including motor neuron loss, gliosis, motor and cognitive deficits, and early mortality [4, 5, 8, 18, 73, 162, 186, 188, 194].

As with the SOD-1 mutants, the TDP-43 rodent models have demonstrated metabolic dysfunction. The conditional neuromuscular knock-out [24, 190] of *Tardbp* in mice led to a reduction in adipose tissue and weight loss [24, 164]. This effect was mediated by depletion of the protein Tbc1 domain family member 1 (*Tbc1d1*), which regulates the translocation of glucose 4 transporter (GLUT4) and energy homeostasis (reviewed in [152]) in skeletal muscles, and which favored lipolysis and leanness. Conversely, TDP-43 overexpression lead to elevated *Tbc1d1* in skeletal muscle, which was associated with an increase in body fat and defective insulin-mediated glucose uptake [161].

A recent study in TDP-43 mutant mice demonstrated the beneficial effects of an energy-rich, high-fat jelly diet in enhancing survival [29]. This was corroborated by the protective effects of high sugar intake in a *Caenorhabditis elegans* model of ALS that expresses mutant TDP-43 [1]. Similarly, supplementation with medium-chain fatty acids and beta-hydroxy butyric acid, as well as genetic manipulation of the carnitine shuttle, which is important to mitochondrial transport of long-chain fatty acids, improves motor functions in a drosophila model of ALS with mutant TDP-43 over-expression [103].

Collectively, evidence from Sod1 and TDP-43 models reveals a potential for metabolic, therapeutic intervention strategies in ALS/FTLD. The next section addresses the critical question of whether and how ALS/FTLD proteins modify metabolism.

## Role of ALS/FTLD-associated proteins in metabolism

ALS/FTLD associated proteins may act directly to regulate cellular and/or whole-body metabolism, which we review here and in Table 2.

**Table 2:** Role of ALS/FTLD associated proteins in cellular or whole body metabolism

Protein	Model	Molecular manipulation	Implication of the protein in metabolic pathways	Citation
TDP-43	Mice	*Tardbp* conditional neuromuscular knock-out	Regulation of obesity-associated gene *Tbc1d1*	Chiang et al. [24]
	Mice	*Tardbp* neuromuscular over-expression	Regulation of glucose transporter GLUT4 translocation	Stallings et al. [161]
	Mice	Knock-in of human hTDP-43^A315T^	Regulation of fatty acid transporter CD36	Stribl et al. [164]
	HCC cell line	*TARDBP* RNAi	Regulation of rate-limiting enzyme of glycolysis PFKP	Park et al. [131]
	iPSC-derived neurons from ALS/FTLD	Over-expression of disease-linked *TARDBP* mutations	Disruption of mitochondrial complex I assembly by TDP-43 pathological mis-localization in ALS/FTLD	Wang et al. [181]
PGRN	Mice	IP injections of recombinant PGRN protein	Control of insulin-resistance, obesity, and adipose tissue dynamics through IL-6 and TNF-α	Zhou et al. [196]
	Mice	IP injections of recombinant PGRN protein	Regulation of insulin sensitivity through PERK-eIF2α axis	Li et al. [90]
TREM2	Mice	*Trem2* over-expression	Regulation of adipogenesis and adipocyte differentiation through Wnt-1/β-Catenin signaling	Park et al. [128, 129]
	Mice	*Trem2* over-expression	Regulation of insulin resistance and hepatic steatosis	Park et al. [130]
FUS	Drosophila	Mutant human *FUS* over-expression	Fragmentation of mitochondria caused by pathological aggregation of FUS to mitochondria	Deng et al. [39]
	N2a cell line	Mutant human *FUS* over-expression	Mutant FUS interacts with enzymes involved in glucose metabolism Regulation of ATP production	Wang et al. [180]
	NSC34 cell line	Mutant human *FUS* over-expression	Regulation of ATP production	Stoica et al. [163]
EWS	Mice	*EWS* knock-out	Regulation of mitochondrial density in pre-adipocytes	Park et al. [128, 129]
C9ORF72	Motor neurons and lymphoblastoid cell lines from ALS patients with C9ORF72 expansion		Regulation of genes involved in cholesterol biosynthesis and glucose metabolism	Cooper-Knock et al. [27]

*Abbreviations*: *TDP-43/ Tardbp* Tat activating responsive DNA binding protein, *PGRN* Progranulin, *TREM2/Trem2* Triggering receptor expressed on myeloid cells 2, *FUS/FUS* Fused in sarcoma, *EWS/EWS* Ewing’s sarcoma, *C9ORF72* Chromosome 9 open reading frame 72, *HCC* Hepatocellular carcinoma, *iPSC* induced pluripotent stem cells, *N2a* Neuroblastoma, *NSC34* Motor- neuron like cells, *RNAi* RNA interference, *Tbc1d1* Tre-2/Bub2/Cdc16 1 domain family member 1, *GLUT4* Glucose transporter 4, *CD36* cluster of differentiation 36, *PFKP* Phosphofructokinase, *IL-6* Interleukin 6, *TNF-α* Tumor necrosis factor alpha, *PERK* Protein kinase RNA-like endoplasmic reticulum kinase, *eIF2α* Eukaryotic initiation factor alpha

### The TDP-43 protein

As noted, TDP-43 can regulate whole-body metabolism and glucose transport through the obesity-related gene *Tbc1d1* [24, 161]. It is now known that the *TARDBP* knock-in mice have increased serum levels of free fatty acids and HDL cholesterol. This phenotype is accompanied by decreased expression of the gene encoding the fatty acid transporter, CD36, and by the dysregulation of a cluster of genes involved in energy production, lipid metabolism, the respiratory electron transport chain and various mitochondrial pathways [164].

In vitro, *TARDBP* regulates glycolysis in a hepatocellular carcinoma cell line through the miRNA-mediated post-transcriptional regulation of a key rate-limiting glycolytic enzyme phosphofructokinase (PFKP) [131]. These studies highlight an important role for TDP-43 in the regulation of key metabolic processes, including glucose and lipid transport and metabolism, and provide a basis for the observed disease-modifying effect of metabolic dysfunction in ALS and FTLD.

### Progranulin

Haploinsufficient, loss-of-function mutations in the human *PGRN* gene are found in 5–20% of patients with familial FTLD and are a common cause of FTLD-TDP ([9, 30, 100]. Progranulin (PGRN) is an ~ 70 kDa, secreted protein that is implicated in numerous cellular processes, from inflammation to wound healing [63, 187]. In the brain, PGRN is believed to function as a neurotrophic factor [150, 174].

Now, PGRN is known to have an important role in lipid metabolism and insulin signaling. In contrast to its pathogenic role in FTLD, *Pgrn* haploinsufficiency in mice confers protection against diet-induced obesity and insulin resistance [120]. Conversely, higher plasma levels of PGRN have been found in patients with T2DM, which is characterized by insulin resistance [139]. Similarly, insulin resistance, adipocyte hypertrophy and obesity result from perturbations in the regulation of the inflammatory cytokines interleukin 6 (IL-6) and tumor necrosis factor α (TNF-α) in mice with increased PGRN through *Pgrn* over-expression or administration of recombinant PGRN [93, 95, 107, 196]. Furthermore, the administration of recombinant PGRN to wild type mice leads to impaired insulin sensitivity [93, 95]. Trehalose, a natural disaccharide, has also been proposed as a possible therapy for FTLD as it can restore wild type levels of progranulin expression in *Prgn* haploinsufficient mice [66]. Taken together, these studies suggest that PGRN lies at the intriguing interface between metabolic disorders and ALS/FTLD, whereby its deficiency is associated with neurodegeneration and its increase is associated with metabolic perturbations.

### The TREM2 protein

A missense variant of triggering receptor expressed on myeloid cells 2 (TREM2) (p.R47H) was recently shown to increase the risk of multiple NDDs, including Alzheimer’s disease and ALS in genome-wide association studies (GWAS). TREM2 is also associated with a poorer ALS prognosis as higher spinal cord levels of TREM2 protein in ALS patients correlate with reduced survival [19]. TREM2 is a 26 kDa transmembrane glycoprotein that is involved in multiple inflammatory processes, including microglia activation [195].

Recent animal studies have revealed that TREM2 can regulate adipogenesis by inhibiting the Wnt10b/β-catenin signaling pathway. This action stimulates adipogenesis by increasing the glycogen synthase kinase-3β-mediated phosphorylation of β-catenin, and by increasing adipocyte differentiation through upregulation of the adipogenic transcription factors CCAAT-enhancer binding protein alpha (C/EBPα) and peroxisome proliferative-activated receptor gamma (PPARγ) [128, 129]. Accordingly, *Trem2* over-expressing mice exhibit insulin resistance, adipocyte hypertrophy, and hepatic steatosis [130]. Similarly to PGRN and TDP-43, the metabolic functions of TREM2 seem to contribute to ALS: TREM2 deficiency is deleterious in ALS, whereas TREM2 over-expression leads to various features of metabolic syndrome that have been shown to be favorable in ALS.

### FET proteins

A family of RNA-binding proteins, called the FET proteins (as the family members include FUS, EWS, and TAF15), is important for RNA metabolism. They form abnormal aggregates in ALS/ FTLD similar to TDP-43 [81, 126, 197]. TAF15 and EWS often co-aggregate with FUS in the neurons and glia in most cases of FTLD-FUS but not in ALS with FUS mutations [101].

As with other FTLD proteins, FUS and EWS may also influence metabolic processes. An analysis of the FUS interactome by mass spectrometry showed that mutant FUS has an increased association with enzymes involved in glucose metabolism, compared to wild-type FUS [180]. Additionally, the over-expression of *FUS* has been reported to significantly reduce ATP levels in human neuroblastoma cells [180]. In HEK293 cells, the accumulation of FUS protein upon *FUS* over-expression reportedly limits Ca^2+^ availability in mitochondria, which is essential for several enzymes of the TCA cycle [58], resulting in decreased production of ATP [163]. In another study, mislocalization of FUS to the mitochondria induced mitochondrial fragmentation in FUS-overexpressing *Drosophila melanogaster* [39], which explained the impaired mitochondrial bioenergetics observed in these flies.

A similar decrease in mitochondrial density is seen in mouse preadipocytes that are deficient for EWS. Moreover, a host of genes involved in mitochondrial respiration and fatty acid β-oxidation were shown to be down-regulated in the liver of EWS knock-out mice [128, 129]. Similarly, EWS has been shown to regulate important metabolism-linked signaling pathways, such as Transforming growth factor- beta (TGF-β) and Insulin-like growth factor- 1 receptor (IGF-1R) [26, 65, 109, 136]. Taken together, these studies suggest that FET proteins, as with TDP43, are involved in energy homeostasis. This hypothesis, however, needs further investigation, especially in vivo.

### The C9ORF72 protein

The discovery of mutations in the chromosome 9 open reading frame 72 (*C9orf72*) gene provided the first genetic and pathogenic link between ALS and FTLD [36, 142]. These mutations result in intronic GGGGCC hexanucleotide repeat expansions, and are present in almost 40% of familial and 10% of sporadic ALS cases characterized by TDP-43 pathology [92]. An exon-array analysis showed that C9orf72 repeat expansions lead to the differentially regulated splicing of several genes involved in cholesterol biosynthesis and glucose metabolism [27]. Recently, C9orf72-based ALS/FTLD rodent models have been generated [94], and it will be interesting to investigate whether alterations in glucose and cholesterol metabolism are associated, in these models, with disease severity.

## Does metabolic dysfunction affect protein aggregation in ALS and FTLD?

**Fig. 1:**
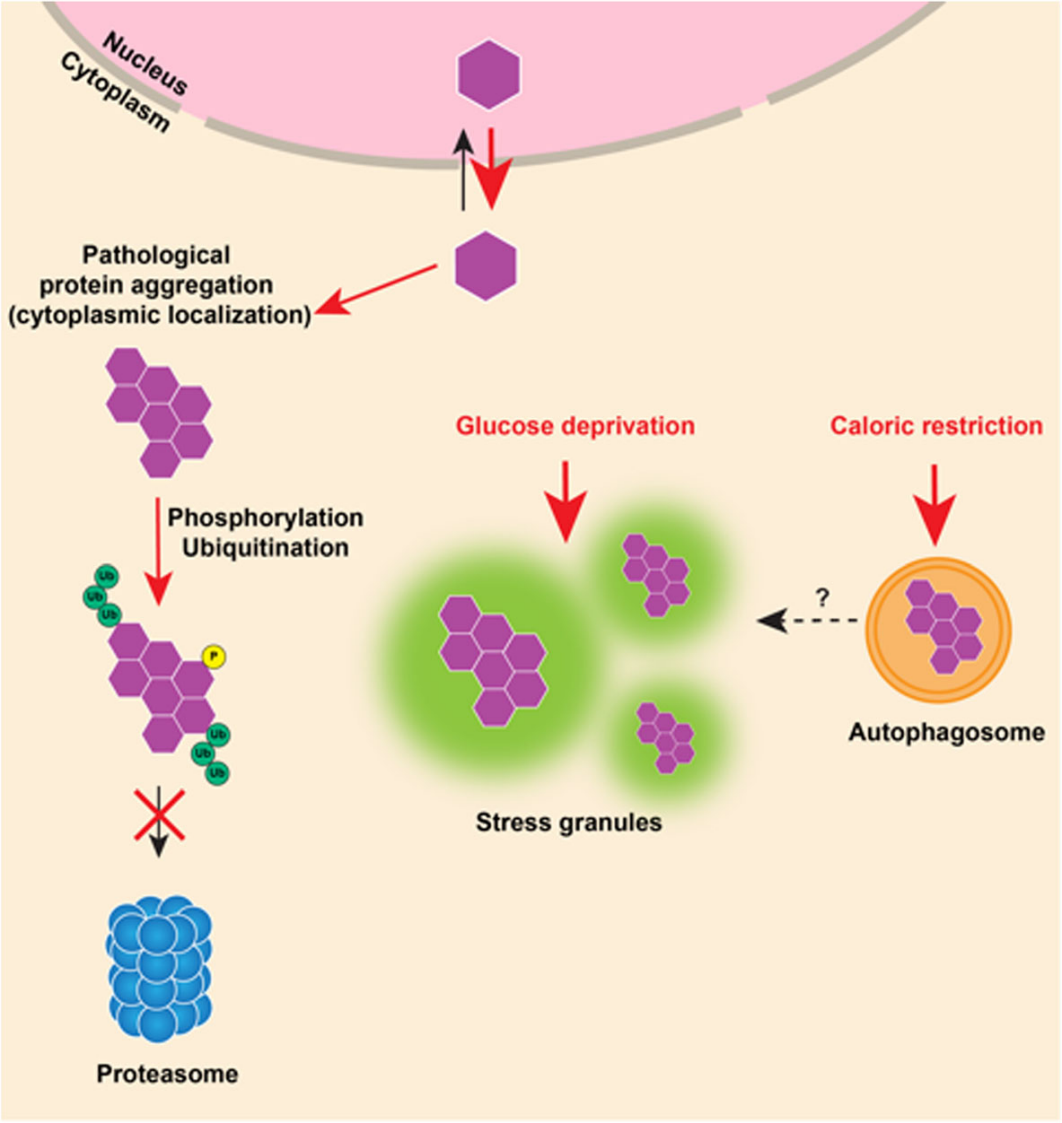
A model of how metabolic processes might contribute to abnormal TDP-43 aggregation in ALS/FTD. TDP-43 is primarily a nuclear protein that mislocalizes to the cytoplasm of neurons in pathological conditions, such as ALS/ FTD, where it undergoes phosphorylation and ubiquitination and forms cytoplasmic aggregates. Glucose starvation might contribute to the formation of TDP-43 aggregates by inducing the formation of stress granules. These stress granules might serve as precursors to TDP-43 inclusions. Similarly, caloric restriction might contribute to TDP-43 aggregation by triggering auto-phagosomes formation, which might serve as another site for TDP-43 aggregation

Both clinical and basic research, then, suggest the involvement of metabolic processes in ALS/FTLD pathophysiology. An vital question to address is whether the abnormal protein aggregation in ALS and FTLD occurs as a consequence of changes in metabolism or vice versa. Here we propose three plausible pathways by which changes in cellular or whole-body metabolism may lead to protein aggregation in ALS and FTLD (Fig. 1).

1. Physiological stress granules have been proposed to act as precursors of abnormal TDP-43 and FUS inclusions in ALS/FTLD [135], supported by the observation that stress granule components co-aggregate with TDP-43 and FUS [14]. Because the formation of stress granules might be induced by glucose deprivation [17], it is conceivable that factors that lead to glucose starvation, such as high-intensity exercise or caloric restriction, might predispose to stress granule formation and subsequently to TDP-43/FUS aggregation in ALS and FTLD. Conversely, T2DM and dyslipidemia might provide protection by supplying more glucose or alternate sources of energy, thereby preventing the formation of stress granules.
2. Changes in the cellular metabolic environment may alter nucleocytoplasmic transport [69], leading to increased localization of TDP-43 or FUS to the cytoplasm, which is a prerequisite step in the formation of their cytoplasmic inclusions. Furthermore, glucose or lipid starvation can lead to activation of AMP-activated protein kinase (AMPK), a major cellular energy sensor, which is found to be active in motor neurons of ALS patients and its activity correlates to the cytoplasmic mislocalization of TDP-43. Consistently, it has been shown that activation of AMPK induces cytoplasmic mislocalization of TDP-43 in motor neuronal cell lines [93, 95]. Rapidly accumulating evidence shows both T2DM and dyslipidemia are associated with reduction in AMPK activity, which was shown to be protective in genetic models of ALS [28, 91].
3. Finally, prolonged caloric/energy restriction induces autophagy in cells [114] might, in turn, enhance protein aggregation. This is supported by the observation that autophagosome markers, such as microtubule-associated protein 1A/1B-light chain 3 (LC3), were present adjacent to TDP-43 inclusions in autopsies from ALS patients [51]. On the contrary, recent evidence suggests that autophagy is reduced in obesity and T2DM. Under diabetic conditions, activation of transforming growth factor beta and miR-192 reduce autophagy in renal glomerular cells [40]. Similarly, high fat diet induced dyslipidemia is known to impair lysosomal functioning and autophagy [195]. Therefore, it is likely that these conditions counteract the formation of autophagosomes, which serve as precursors to pathological aggregates in ALS/FTLD.

## Does altered metabolism counteract effects of protein aggregation in ALS/FTLD?

**Fig. 2:**
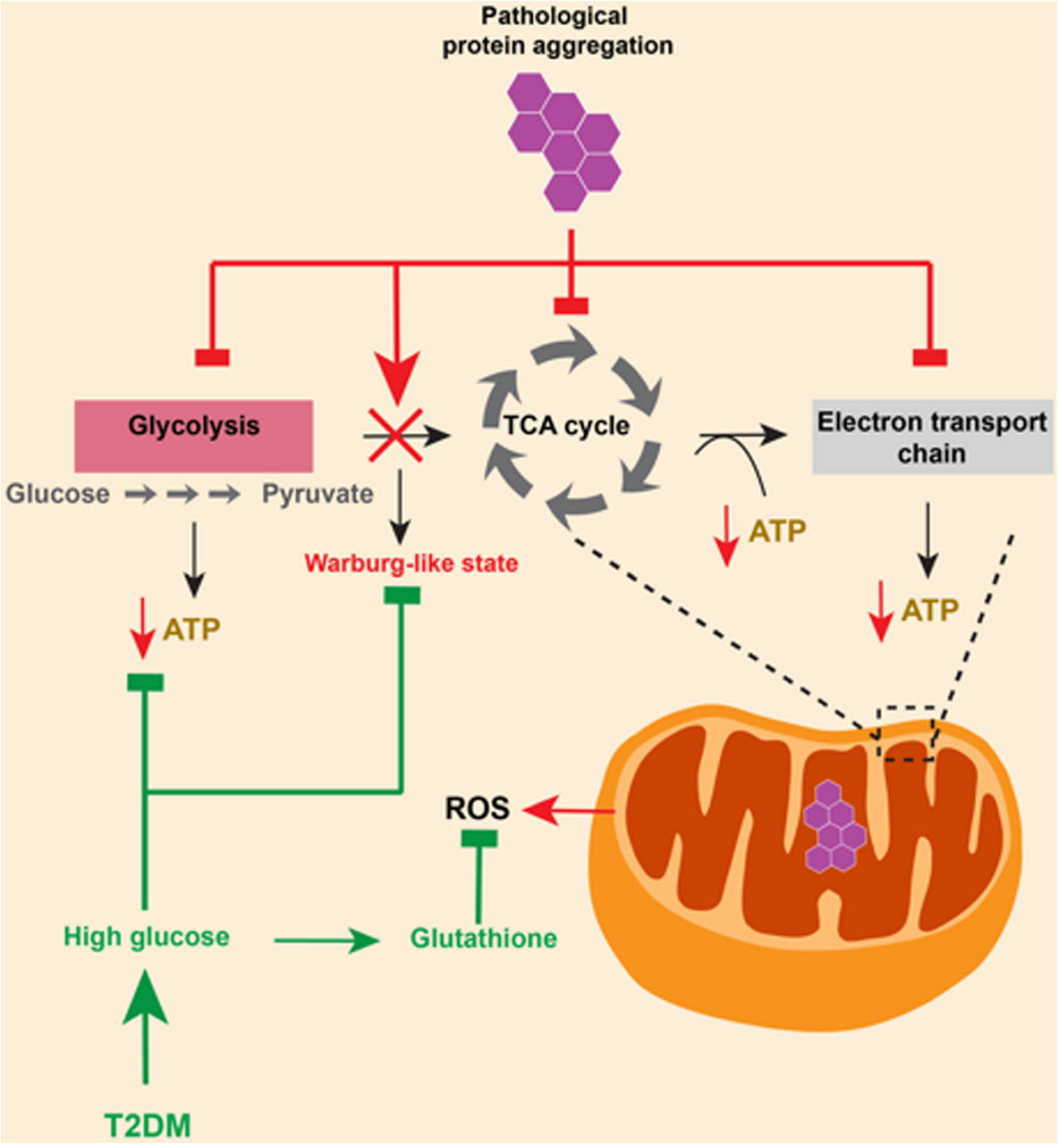
TDP-43 aggregation and defective neuronal glucose metabolism. Loss of TDP-43 function, caused by its abnormal aggregation in neurons, can potentially lead to defective glycolysis and to defects in the TCA cycle or electron transport chain, leading to decreased ATP levels. Additionally, TDP-43 loss of function might induce a Warburg effect in neurons, increasing their reliance on glycolysis for ATP production. Finally, TDP-43 aggregation can lead to increased production of reactive oxygen species (ROS). Type 2 diabetes mellitus (T2DM) can be beneficial in each of these pathogenic mechanisms; hyperglycemia in T2DM provides readily available fuel for glucose metabolism and can also meet the increased requirement of glucose in neurons as a result of the Warburg effect, and it can counter ROS by replenishing levels of the anti-oxidant glutathione

An alternate possibility is that the abnormal protein aggregation in ALS and FTLD causes neurons to become unusually sensitive to metabolic deprivation, in a way that renders metabolic perturbations, such as dyslipidemia and T2DM, beneficial (Fig. 2). There are three major ways in which this could occur:

1. TDP-43, and other proteins that aggregate in ALS and FTLD, could be potent regulators of core cellular metabolic processes. As such, their loss of function through aggregation might lead to a state of cellular ATP deficit. Indeed, there is evidence to suggest that TDP-43 regulates glycolysis in hepatocellular carcinoma cell lines [131], and one study demonstrated that the abnormal localization of TDP-43 to mitochondria in cells from ALS/FTLD patients leads to the disassembly of the respiratory chain complex I and impairs mitochondrial oxidative phosphorylation [181]. This has been corroborated by two more studies showing a pathological displacement of TDP-43, or its N- and C-terminal fragments, in mitochondria leading to impairment of mitochondrial functioning [34, 153]. Hyperglycemia could counteract this by providing more substrate (glucose) for cellular glucose metabolism, thereby partially replenishing the ATP deficit.
2. Another possibility is that loss of TDP-43 function (and/or of other ALS- associated proteins) induces a Warburg-like state [176] in neurons, causing neurons to rely on readily available glucose to support their enhanced glycolysis. Hyperglycemia in T2DM might thus also help to offset the upregulated glycolysis caused by this metabolic maladaptation.
3. Finally, high levels of glucose and/or lipids may counteract the cellular toxicity that arises from abnormal protein aggregation in ALS/FTLD neurons. Both wild-type TDP-43 and FUS regulate the expression of anti-oxidative genes, and their loss of function may lead to increased cell toxicity by reactive oxygen species (ROS) [115, 154]. Other evidence suggests that mitochondrial respiration is decreased in skeletal muscles of patients with T2DM [112]. This decrease in mitochondrial respiration could directly aid in reducing the toxicity due to ROS by reducing their production.

In conclusion, there is a plausible biological rationale for the disease-modifying effect of metabolic disorders in ALS and FTLD based on the aforementioned hypotheses. How about other NDDs?

## Metabolic dysfunction and other neurodegenerative disorders

The disease modifying effects of altered metabolism may not be limited to ALS and FTLD: they may be important for other NDDs [10, 137].

Huntington’s disease (HD) is a hereditary NDD caused by elongated CAG repeats in the Huntingtin (*Htt*) gene that lead to progressive increases in choreic movements and to neuropsychiatric dysfunction [146]. It is characterized by reduced mitochondrial ATP production in patient-derived lymphoblasts and skeletal muscles [96, 151, 156]. Importantly, a high BMI at the time of HD onset is associated with slower disease progression [117].

Similarly, patients with advanced Parkinson’s disease (PD), a movement disorder marked by ɑ-synuclein aggregates in neurons [87], exhibit unintended weight loss [14, 21], which may occur as a consequence of increased resting energy expenditure [89, 106]. Interestingly, similar to ALS, pre-morbid T2DM slows the progression of PD [31, 99].

Finally, metabolic factors also modify the course of Alzheimer’s disease (AD), the most common form of late-onset dementia, which is characterized by synaptic and neuronal loss and by the formation of β-amyloid plaques and neurofibrillary tau tangles in neurons [178]. While obesity in mid-life is associated with an increased risk of AD [85, 184], a higher BMI later in life, and slower BMI decline after onset of AD and slow disease progression [71].

## Conclusions and future directions

In conclusion, the current clinical literature suggests that metabolic factors, such as BMI, diet and exercise, and metabolic disorders, such as T2DM and dyslipidemia, have a disease-modifying effect in ALS and potentially in FTLD (Table 1). These disease-modifying effects may be linked to the involvement of ALS and FTLD-related proteins in cellular and whole body metabolism (Table 2). However, altered metabolic pathways may also stimulate the formation of characteristic protein aggregates in ALS and FTLD (Fig. 1) or could modulate their downstream toxicity (Fig. 2).

It is imperative that future clinical studies of these diseases are carefully designed to reach conclusive answers. Studies on the effect of T2DM, dyslipidemia and BMI on ALS disease course should be prospective and should adjust for their individual and combined effects, as T2DM, dyslipidemia, and obesity are often co-morbid. Additional confounding factors, such as gender, ethnicity, diet, smoking, other vascular co-morbidities, and medications should be accounted for. It will also be important to correlate parameters of disease progression in ALS (e.g., the ALS-FRS) to those in metabolic disorders (e.g., serial measurements of HbA1c and lipid profile). Furthermore, it will be crucial to assess cognitive function when studying the effects of metabolic disorders on ALS because there may be different effects of metabolic disorders on motor vs. cognitive parameters [75, 76]. Finally, the disease-modifying effects of T2DM, dyslipidemia and BMI should be studied in more detail in patients with FTLD.

The recent development of autopsy brain banks for NDDs provides an excellent resource with which to study the effects of metabolic factors and dysfunction on the neuropathological pathways affected by ALS and FTLD. Through carefully designed clinicopathological studies, the extent and distribution of TDP-43 pathology in specific brain regions may be compared between ALS/FTD patients, who have or do not have a particular metabolic disorder. Similarly, a comparison of downstream pathways, such as those involved in neuronal loss, astrogliosis, autophagy, microglial activation, mitochondrial respiration, AMPK activity, and ROS levels, might provide important clues as to whether the effects of metabolic disorders on ALS/FTLD occur prior to or after protein aggregation occurs.

Most studies that have investigated a role for metabolic factors in the pathology of ALS/FTLD were performed in vivo in animal models, which have supported and advanced clinical findings in humans, where it is more difficult to control for various factors. Immortalized cancer cell lines and in neural stem cells are also excellent models to study; however, they can show metabolic adaptations that complicate interpretation of data derived from them [23, 68]. Other systems that may be excellent for studying metabolic effects in real time include alternative ex vivo systems, such as organotypic slice culture models [88], induced pluripotent stem cells (iPSCs)-derived neurons [108] or long-term complex neuronal cultures [159]. At the same time, they allow for genomic, transcriptomic, proteomic, metabolomics, and epigenetic analyses. Finally, we need to better understand how the nuclear localization, aggregation, and phosphorylation of TDP-43 and FUS might be influenced by altered metabolic states, such as hyperglycemia and dyslipidemia, and how these states might protect neurons from damage. We also need to experimentally investigate the role of astrocytes and microglia in the potential disease-modifying effect of metabolic disorders in ALS and FTLD.

## Conclusion

In conclusion, we have presented hypotheses that bridge the basic and clinical lines of research related to the disease-modifying effect of metabolic factors and disorders in ALS/FTLD. This review may serve as an inspiration for translationally oriented studies into the topic, which has important implications for prevention and treatment of ALS/FTLD.

## Abbreviations

ALS: Amyotrophic lateral sclerosis
BMI: Body mass index
C9orf72: C9 open reading frame 72
EWS: Ewing’s sarcoma
FTD: Frontotemporal dementia
FTLD: Frontotemporal lobar degeneration
FUS: Fused in sarcoma
GLUT4: Glucose transporter 4
HDL: High density lipoproteins
LDL: Low density lipoproteins
OR: Odds ratio
SOD1: Superoxide dismutase 1
T1DM: Type 1 diabetes mellitus
T2DM: Type 2 diabetes mellitus
TDP-43: TAR DNA activating protein of 43 kDa
